# Clinical relevance of blood-based ctDNA analysis: mutation detection and beyond

**DOI:** 10.1038/s41416-020-01047-5

**Published:** 2020-09-24

**Authors:** Laura Keller, Yassine Belloum, Harriet Wikman, Klaus Pantel

**Affiliations:** University Medical Center Hamburg-Eppendorf, Institute of Tumor Biology, Martinistrasse 52, Building N27, 20246 Hamburg, Germany

**Keywords:** Molecular medicine, Biomarkers, Oncology

## Abstract

Cell-free DNA (cfDNA) derived from tumours is present in the plasma of cancer patients. The majority of currently available studies on the use of this circulating tumour DNA (ctDNA) deal with the detection of mutations. The analysis of cfDNA is often discussed in the context of the noninvasive detection of mutations that lead to resistance mechanisms and therapeutic and disease monitoring in cancer patients. Indeed, substantial advances have been made in this area, with the development of methods that reach high sensitivity and can interrogate a large number of genes. Interestingly, however, cfDNA can also be used to analyse different features of DNA, such as methylation status, size fragment patterns, transcriptomics and viral load, which open new avenues for the analysis of liquid biopsy samples from cancer patients. This review will focus on the new perspectives and challenges of cfDNA analysis from mutation detection in patients with solid malignancies.

## Background

Cell-free DNA (cfDNA) refers to extracellular DNA molecules (double-stranded DNA and mitochondrial DNA) originating from any cell type found in body fluids. cfDNA has been detected in the blood of diseased and healthy individuals already in 1948.^1^ cfDNA analysis is currently applied in prenatal diagnostics^2^ and its clinical use is also evaluated in several fields including cancer, organ transplant, autoimmune diseases, trauma, myocardial infarction and sepsis.^3–7^ However, our understanding of the structure and origins, cell release mechanisms and clearance of cfDNA is still preliminary. Although the majority of cfDNA molecules originate from the haematopoietic system, there is a huge interest to determine the relative contribution of different organs in healthy and pathological conditions to the overall amount of cfDNA. Not only a multitude of release mechanisms including apoptosis, senescence, ferroptosis, NETosis, phagocytosis and necrosis, but also active secretion—including association to extracellular vesicles or induced by other mechanisms like expulsion of mature nuclei by erythroblasts, egestion of mitochondrial DNA or vital NETosis—have been described. On the other side, diverse parameters govern the degradation and elimination of cfDNA molecules: enzymatic cleavage in the circulation, elimination of nucleosome complexes by the liver and to a lesser extent removal of DNA fragments by the kidney. The description of these fundamental aspects of cfDNA biology is out of scope of this introduction, but has been discussed in excellent comprehensive reviews.^8,9^

The tumour-derived fraction of cfDNA, commonly named circulating tumour DNA (ctDNA), has received enormous attention during the last decade owing to its huge potential as a minimal invasive tumour biomarker in cancer patients. As for cfDNA, the correlation between tumour biology and ctDNA release is still not well understood and may not solely rely on the amount of dying cells. Not only the volume and metabolism of the tumour, but also its rate of proliferation, have been positively correlated to the amount of ctDNA in blood plasma.^10–12^ Nevertheless, the proportion of ctDNA engulfed into extracellular vesicles actively released by tumour cells is still unclear and the effect of different therapy regimens on this active secretion mostly unknown.^13,14^ Obviously, there is a huge need for more fundamental research on the kinetics of ctDNA in cancer patients.

**Fig. 1:**
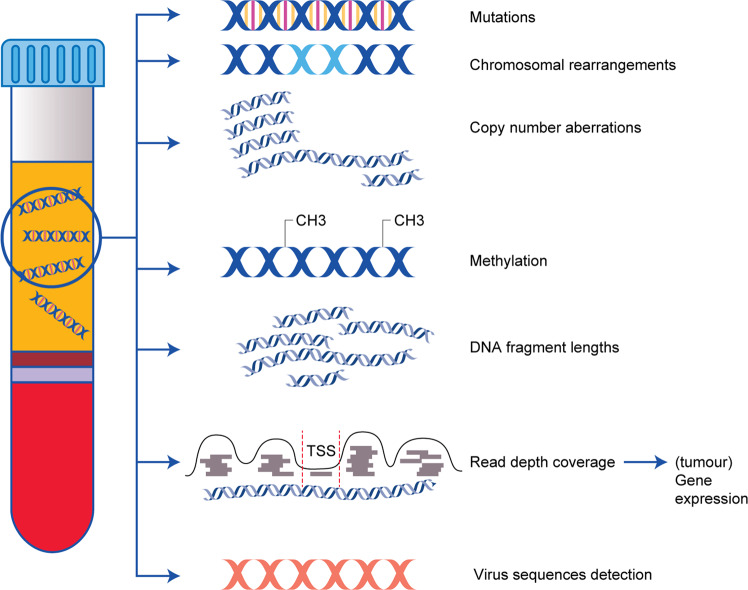
Different features of ctDNA and potential clinical implications. This figure summarises the tumour-relevant clinical information that can be gained from the study of different features of cfDNA. Somatic genomic aberrations detectable on ctDNA include mutations, chromosomal rearrangements and copy number aberrations. Additional features characteristic for ctDNA are specific epigenetic aberrations like methylation patterns or different DNA fragment lengths. Information on tumour-specific transcription can also be obtained from ctDNA analysis by reading the inter-nucleosome depth coverage. In virus-induced tumours (e.g. EBV-related nasopharyngeal carcinomas or HPV-related head and neck tumours), the quantitative assessment of virus sequences have diagnostic validity. TSS transcription starting sites.

The vast majority of published studies on the potential use of ctDNA in oncology deal with the detection of specific mutations detected in plasma or serum of cancer patients, and these studies have been reviewed in detail elsewhere.^7,15^ Briefly, mutation detection in ctDNA has the potential to be used in early cancer detection, to determine the tissue of origin, prognosis, to monitor response and assess potential resistance to the treatment, or to detect minimal residual disease. However, epigenetic alterations are even more frequent than somatic mutation in cancer development.^16^ Although mutation analysis of ctDNA shows a number of clinical applications, the assessment of cfDNA beyond the detection of point mutations, encompassing the study of chromosomal rearrangements, copy number aberrations, methylation, fragmentation and gene expression, is therefore also receiving increasing interest (Fig. 1).

Obviously, some tumour types and body sites release lower amounts of ctDNA into the bloodstream. Here, non-blood sources of ctDNA for molecular profiling have become valuable. Clearly in primary brain tumours, such as gliomas, central nervous system lymphomas and some paediatric solid tumours, cerebrospinal fluid (CSF) has shown higher sensitivity compared with peripheral blood.^17,18^ Similarly, for some upper aerodigestive track tumours saliva, sputum or pleural effusions may also be good alternatives to blood, with recent reviews published elsewhere.^19,20^ Urine, stool and seminal fluid are other examples of body fluids that have been used in different liquid biopsy approaches.^21^

Here, we will focus on ctDNA detected in blood plasma of cancer patients. We begin this review by providing an overview of the main methods used to detect mutations in ctDNA before discussing some of the associated challenges; it is not our aim, however, to comprehensively cover this topic within this review. We will then outline additional features of cfDNA beyond the detection of point mutations that can be assessed using liquid biopsy samples from patients with solid tumours.

## Mutations in ctDNA

### Approaches for the mutation analysis of ctDNA

Mutations in ctDNA from liquid biopsy samples can be detected via two different approaches. In the first approach, single, or low numbers of, mutations can be detected using highly sensitive techniques with high specificity and at a rather fast and cost-effective rate.^22^ In 2016, the Cobas EGFR mutation Test v2 that interrogates by RT-PCR several mutations in exons 18, 19, 20 and 21 of epidermal growth factor receptor (*EGFR*) gene was the first liquid biopsy-based companion diagnostic to be approved by US Food and Drug Administration (FDA) and the European Medicines Agency for the prescription of EGFR inhibitors in patients with non-small-cell lung cancer (NSCLC) in cases when tumour biopsy tissue is not available.^23^ Other targeted approaches, based mainly on digital PCR (droplet digital [ddPCR] or BEAMing dPCR), have been demonstrated to be able to detect specific known mutations, such as the main driver mutations of the primary tumour or variants associated with response to drugs in individual tumour types, and usually show high concordance with results obtained in tumour tissue^24–26^ and reach a variant or mutant allele frequency detection (VAF/MAF) as low as 0.001% for the most advanced technologies^27^ (i.e. the frequency of a particular genetic variation of a specific sequence [e.g. allele/mutation] relative to the other genetic variations of the same sequence). The detection and comprehensive molecular characterisation of minimal residual disease (MRD) is of particular importance in the adjuvant setting to improve clinical outcomes;^28^ ctDNA detected via such targeted, highly sensitive approaches in the early stages of melanoma was reported to predict the relapse risk,^29,30^ and might therefore be useful in the process of patient stratification for adjuvant therapy. Next step in the implementation of ctDNA in clinical routine is to demonstrate its utility in patient treatment selection. For instance, in the recently published TARGET study (registered in NIHR Central Portfolio Management System under the reference CPMS ID 39172), the primary aim was to match advanced stage patients to early phase clinical trials on the basis of plasma ctDNA analysis of both somatic mutations and copy number alterations in 641 cancer-associated-genes.^31^ Another example is the Circulating Tumour DNA Guided Switch (CAcTUS) study (NCT03808441), which determines whether switching from targeted therapy to immunotherapy based on a decrease in levels of ctDNA in the blood will improve the outcome in melanoma patients.

Broader approaches have also been developed to interrogate multiple mutations in parallel and range from the analysis of several tens of mutations, to a genome-wide analysis of cfDNA by whole-exome sequencing (WES) or whole-genome sequencing (WGS). Most of these approaches use next-generation sequencing (NGS) but mass-spectrometry-based detection of PCR amplicons is also becoming available.^32^ Besides increasing the probability of detecting a mutation in cfDNA, these broader approaches allow a more complete genotyping of the tumour, which can be used to assess tumour heterogeneity or to follow clonal evolution of the tumour under treatment, as well as to identify potential resistance mutations before clinical progression is observed.^10,33,34^ Another example of the application of nontargeted approaches also relates to cancer patients treated by immunotherapy, for whom mutation load (i.e. the number of nonsynonymous mutations found in a tumour) has emerged as a putative biomarker of the response to the treatment. Assessing mutation load and measuring its evolution through plasma analysis has also been evaluated as an alternative approach to tumour tissue determination.^35,36^ More generally, comprehensive reviews have discussed the clinical utility of ctDNA in the new era of immunotherapy.^37,38^

However, one should be aware that the larger the panels, the more expensive and difficult it is to obtain high sensitivity for mutation calling.

### Challenges associated with mutation detection in cfDNA

A key issue in the analysis of ctDNA is still the extent to which the information gained from the liquid biopsy sample reflects the tumour tissue. Both technical and biological factors can affect the concordance between tumour and plasma, generating false-negative and false-positive results in ctDNA analysis.

False-negative results might be explained by the low volume of plasma yielded (4–5 ml) from a typical blood sample of 10 ml, which limits the total number of available genome copies to be analysed: mutations within a tumour can be clonal or subclonal, and the amount of available genome copies is a limiting factor for the detection of variants of low allele frequency.^39^ Moreover, the tumour fraction of cfDNA varies between cancer types as well as between patients affected by the same cancer type.^40^ Even at the metastatic stage, some patients can yield a low amount of ctDNA,^41,42^ and the question of why some tumours undergo limited shedding of ctDNA is still not completely resolved. In this regard, detection of mitochondrial tumour-derived DNA, as an alternative source of ctDNA might be a promising approach, owing to the thousands of copies of mitochondrial DNA per cell.^43^ Proof of principle for this apporach was provided in patient-derived orthotopic xenograft models of glioblastoma in 2019.^11^ Considerations about technical improvements for the methods used to analyse cfDNA could also help to overcome the limit of detection. Ultra-deep sequencing methods can lower the percentage of false negative and are currently under evaluation across different cancer types.^44–47^ The size selection of cfDNA fragments (see below) or the choice of an alternative method for library preparation like single strand DNA libraries for NGS are additional solutions.^48^

False-positive results are another concerning issue when multiple mutations are interrogated by NGS platforms. The risk of introducing errors during library preparation and subsequent sequencing steps has led to the implementation of multiple mutation-enrichment methods and error-suppression strategies such as the introduction of molecular barcodes or bioinformatic analysis pipelines of the data.^22,39,49^ The extensive comparison of paired tumour and plasma samples therefore represents an important prerequisite to evaluate the diagnostic accuracy of analytical platforms, especially for variants with allele fractions that are close to the limit of detection.^50–52^ Different commercial NGS platforms might not have the same limit of detection or interrogate the same genomic regions as each other, and the field would benefit from rigorous cross-assay comparisons, as carried out between 2015 and 2019 by the EU Innovative Medicines Initiative (IMI) consortium CANCER-ID (www.cancer-id.eu) and sustained by the new European Liquid Biopsy Society (ELBS; www.elbs.eu) and other networks (the US Blood Profiling Atlas of Cancer; www.bloodpac.org). A cross-comparison of four commercial NGS platforms, all certified by the US-based college of American Pathologists-Clinical Laboratory Improvement Amendments, was carried out in 2019 with plasma–tumour-matched samples of early stage cancers that present a limited ctDNA amount.^53^ Substantial variability in terms of sensitivity (38–89%) and positive predictive values (36–80%) was identified among the different platforms. Low predictive positive values were mainly associated with variants with an allele frequency below 1% and could be explained by technical factors (limited sensitivity, bioinformatic filtering of the data or even plain error of identification). Nonetheless, germline variants shed from normal cells and during clonal haematopoiesis (e.g. the presence of somatic variation in some cancer-related genes like *TP53* that do not necessarily lead to cancer) constitute another source of confounding factors that have to be considered when interpreting the data. By applying a highly sensitive and specific ctDNA sequencing assay on a cohort of 124 metastatic cancer patients and 47 controls without cancer, with matched white blood cell DNA, Razavi et al. found that 53.2% of mutations found in cancer patients had features consistent with clonal haematopoiesis.^47^ This study highlights therefore the risk of false findings and the need to integrate white blood cell DNA as control when applying ultrasensitive ctDNA sequencing methods. Overall, it appears necessary that laboratories should comment on these different limitations in their reports.^54^

If these technical and biological factors could be ruled out, then ctDNA could be used to evaluate intratumour heterogeneity, as it is now well accepted that a single tumour biopsy procedure generates a limited representation of temporal and spatial heterogeneity, whereas ctDNA in plasma would represent a pool of the entire tumour or of the metastatic sites.^55^ Up until now, clinical studies that have compared plasma analysis with  multiregional tissue biopsies are rare and limited to few patients, due to an increase risk of clinical adverse side effects linked to this invasive procedure (see Table 1). In this sense, studies conducted utilising rapid autopsy programs are of particular interest.^26^ Some studies have shown that the quantitative level of mutations found in ctDNA reflects the architecture of the mutational landscape in tumour tissue, with truncal mutations more readily detectable than private mutations.^10,56–58^ In the context of acquired resistance in gastrointestinal cancers, mutation analysis of ctDNA taken at progression was more informative than the corresponding analysis of tissue biopsies.^34^ However, in some cases of melanoma patients ctDNA analysis only partially reflected heterogeneity, with under-representation of certain anatomical metastatic sites like brain or subcutaneous metastases.^12^ A better understanding of the parameters that govern ctDNA release (i.e. proliferation/turnover, active secretion, type of cancer, location or tumour vascularity) is therefore needed.

**Table 1:** Studies evaluating the capacity of ctDNA to recapitulate intratumour heterogeneity.

Cancer entity	No. of patients	No. of tumour biopsies per patient	Tissue sequencing technique	cfDNA sequencing technique	Time of plasma collection	Concordance and conclusions	Reference
Metastatic serous ovarian cancer	1	8 collected at initial diagnosis/surgery	Tam sequencing	Tam sequencing	Plasma samples were collected 15 and 25 months after initial surgery	TP53 was identified in 8/8 tissue biopsies at initial surgery. EGFR and TP53 mutations were found in plasma samples. Trace signal of the EGFR mutation in 2/8 tumour biopsies obtained from same metastasis, using a lower-specificity criteria defined for mutation detection.	^143^
Metastatic breast and ovarian cancer	1	1 tissue sample from breast and 4 ovarian tissues	Shotgun massive parallel sequencing	Shotgun massive parallel sequencing	Plasma samples were collected at diagnosis and 1 day after the operation.	SNV found in tumour were classified into seven different groups according to the degree of sharing these mutations between the four regions. Mutations that were shared by all four regions contributed the highest fractional contribution of tumour-derived DNA to the plasma. Mutations that were more region specific had a reduced contribution to plasma.	^56^
Metastatic breast cancer	1	8 tumour biopsies obtained at diagnosis from primary tumour and an LN; after 19 months from the brain metastasis; at autopsy breast, chest, liver, ovary and vertebrae.	WES confirmed by deep sequencing	WES confirmed by deep sequencing.	9 serial plasma samples collected during the last 500 days of clinical follow-up.	In plasma, trunk mutations from tumoural tissues were highest in abundance whereas metastatic-clade mutations were lower in abundance Plasma DNA captured differential response across distinct metastatic sites during targeted treatment 11 nonsynonymous high-confidence SNVs were identified and validated in plasma but not detectable at >2% AF in any of the analysed tumour biopsies. Among these, one was associated with resistance to treatment	^57^
Metastatic breast cancer	1	Primary tumour and 1 synchronous liver metastasis	NGS panel of 300 genes known to harbour actionable mutations	NGS panel of 300 genes known to harbour actionable mutations	Plasma samples were collected before therapy, and during at 2 and 6 months and at progression.	All plasma samples captured the entire repertoire of mutations found in the primary tumour and/or metastatic deposit	^144^
Metastatic colorectal cancer	1	primary sigmoid tissue and 2 liver metastases	Amplicon based sequencing (17 mutations)	ddPCR on RAS pathway hotspot mutations	Plasma was collected every 4 weeks until disease progression.	4/7 of tumour tissue mutations were identified in plasma	^145^
Metastatic gastro- intestinal cancer	5	Between 3 and 17 biopsies/ patient	Targeted exome sequencing	Targeted NGS panels (70 genes or 226 genes) Some SNV were confirmed with ddPCR	Plasma and tissue were obtained in parallel at progression and at rapid autopsy	Tumour biopsy identified resistance alterations less frequently than cfDNA.cfDNA detected multiple resistance alterations residing concurrently in distinct tumour subclones and different metastatic lesions.	^34^
Metastatic NSCLC	1	12 (7 metastatic and 5 primary tumour regions)	WES	Bespoke targeted NGS panels (103 variants)	5 PT regions were obtained at diagnosis, 1 metastasis during treatment (day 467) and 6 metastases at autopsy. 9 plasma samples were analysed during follow-up (day 151, 242, 340, 431, 466, 627. 662, 767).	At day 466, 18 out of 20 SNVs were detected in ctDNA; these subclonal clusters were shared between six out of seven metastatic sites. Single SNVs from two private subclones were also detectable in but were not identified vertebral biopsy. ctDNA analysis also identified 90 days before death subclones private to one metastatic site that was not identified in CT scan.	^10^
Surgical resectable NSCLC	32	181 multi-region tumour tissues in total were analysed	Targeted capture sequencing (1021-gene panel)	Targeted capture sequencing (1021-gene panel)	Not mentioned	Much easier to detect trunk mutations than branch mutations in ctDNA	^58^
Stage I–III NSCLC	4	Between 2 and 3 biopsies/patient	50 SNV Multiplex PCR-NGS	50 SNV Multiplex PCR-NGS	Plasma samples were collected prior to surgical resection of tumours.	43% of the selected mutations were detected in both cfDNA and tumour DNA, 25% of which were variants occurring late during tumour evolution and predicted to be subclonal in origin.	^146^
Metastatic gastric cancer	5	5	Customised 483 genes panel	Customised 483 genes panel	Blood samplesand tumour tissue samples were collected simultaneously.	The numbers of somatic SNVs and InDels in the plasma samples differed from those of the biopsies. The mutated genes identified in the plasma were all detected in one or more biopsy, which demonstrated that plasma ctDNA could partially overcome tumour heterogeneity	^147^
Metastatic melanoma	3	3 or 4 biopsies/patient	WES	WES	Plasma samples were collected at disease progression, and tissue samples were collected at death	99% ubiquitous mutations (present in all tumours), 64% shared mutations (present in two or more tumours), and 14% private mutations (present in only one tumour) were identified in plasma. Under-representation of ctDNA from subcutaneous disease sites and brain. Limited ability to detect private mutations in plasma was a result of the low mutant allele frequency.	^12^

*ddPCR* droplet digital PCR, *EGFR* epidermal growth factor receptor, *LN* lymph node, NGS next-generation sequencing, *SNV* single nucleotide variant, WES whole-exome sequencing.

## Copy number and structural DNA aberrations

As well as mutations, other cancer-related alterations in DNA (such as copy number aberrations [CNA]) and genomic rearrangements (inversions, translocations, insertions and deletions) can be studied using cfDNA. CNA can now also be easily detected by massively parallel sequencing methods thanks to the development of diverse analytical tools based on different features that can be extracted from NGS data (reviewed in ref. ^59^). CNA are estimated to be present in almost all cancers of most histopathological types, so that the detection of CNA in cfDNA could potentially facilitate noninvasive diagnostic applications. However, the identification of CNA in cfDNA has proven challenging due to the prevalence of copy number variation in the healthy population,^60^ the variable level of the tumour fraction in cfDNA, tumour ploidy and tumour heterogeneity. Currently, CNA in cfDNA can be detected using low-coverage (0.1×) sequencing of the genome followed by normalisation algorithms; this approach necessitates a ctDNA fraction above 5% to achieve good specificity and sensitivity,^56,61–63^ although targeted approaches and new algorithms to detect CNA in a lower amount of ctDNA (below 1%) have been developed within the past 5 years.^64,65^ Again, whether CNA detected in plasma are representative of the tumour tissue is still a subject of investigation. In patients with hepatocellular carcinoma, CNA in plasma were comparable with respect to their size profile, with those found in tumour tissue in 63% of the chromosome arms analysed.^66^ In 2018, a new algorithm for aneuploidy detection based on the amplification of long interspersed nuclear elements (LINEs) was evaluated on a large cohort of plasma samples from early and late stages of eight different cancer types that presented with a variable neoplastic cell fraction. Fifty-four percent of plasma samples had a concordant gain or loss in the primary tumour.^65^ The presence of CNA in plasma has also been associated with clinical outcome, and their analyses have revealed new resistance mechanisms in patients with prostate cancer or NSCLC such as androgen receptor (AR) amplification and TMPRSS2‐ERG fusion or MYC amplification, respectively.^67,68^

Genomic rearrangements, notably those involving the genes encoding the kinases ALK or ROS, or the presence of the fusion TMPRSS2-ERG, are potential therapeutic targets in lung cancer or a sensitivity biomarker for abiraterone acetate treatment response in prostate cancers, respectively.^69,70^ These structural genomic abnormalities have the potential to be detected via NGS techniques with the additional benefit of detecting a large number of gene fusions with known and unknown partner genes, compared with previous targeted PCR assays. Indeed, data obtained over the past 1–2 years have shown that plasma genotyping using hybrid-capture NGS technology can reliably detect ALK or ROS fusions in NSCLC patients,^71,72^ although these results need to be confirmed in larger patient cohorts.

## DNA fragmentation patterns

**Fig. 2:**
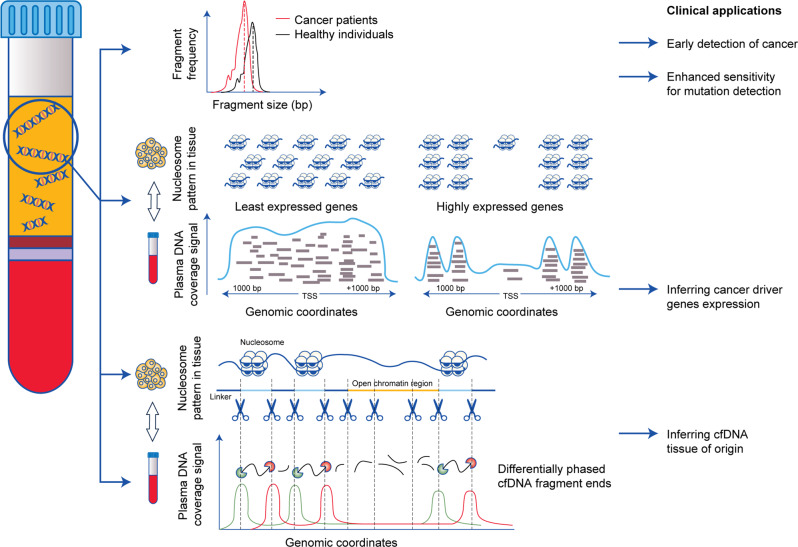
Clinical applications of genome-wide fragmentation analysis of cfDNA in cancer patients. Analysis of length distribution of cfDNA has revealed that cancer patients present a more fragmented pattern (and consequently shorter fragments) than healthy donors. This feature can be leveraged to detect cancer without previous knowledge of genomic aberration but also to enhance sensitivity of mutation detection when monitoring tumour evolution. cfDNA coverage signal around TSS correlates with gene expression. Actively transcribed promoters at TSS display low nucleosome occupancy (that is translated in very low read numbers of cfDNA fragments) flanked by well-phased nucleosomes (translated in relatively high and well-phased read numbers of cfDNA fragments). Nonetheless, the region around an active TSS exhibits an overall lower coverage in comparison to inactive TSS promoters, which exhibit an increased coverage signal indicative of denser nucleosome packaging. Therefore, unravelling nucleosome occupancy at promoters from plasma DNA sequencing might help inferring expression levels of genes in the contributing cell types. cfDNA fragment ends pattern reflects nucleosome-depleted region and well-phased nucleosome arrays around the tissue-specific open chromatin region. This analytical approach allows by comparison of nuclear DNA from tissues for the determination of the relative contributions of various tissues in plasma DNA. For the design of our Figure, we were inspired by the figures in the publications of Van der Pol et Mouliere^8^ and Murtaza et Caldas.^142^

Several different studies published over the past 20 years have focused on the size fragmentation pattern of cfDNA, i.e. the length distribution of cfDNA fragments, which reveals relevant genetic ‘non-coding’ clinical information (Fig. 2). cfDNA size profiling is a fundamental parameter that can contribute to the better definition and detection of ctDNA. Not only does cfDNA size profiling provide clues about the origins of ctDNA, but it can also provide further clues about how to improve the analytical methods.

### cfDNA fragmentation pattern analysis for better definition and detection of ctDNA

Gel electrophoresis and electron microscopy were first used to analyse the length of cfDNA in the plasma or serum of cancer patients and healthy donors and revealed that plasma DNA is not randomly fragmented. Fragments equivalent to whole number multiples of 180 bp^73,74^ were first observed in both cohorts; however, different size distributions between healthy donors and cancer patients were already observed.^73^ This figure was further refined to ~160 bp using NGS methods,^66^ a result that inferred the existence of a nucleosome footprint and suggested that the release of DNA by apoptotic caspase-dependent cleavage was a major contributor to cfDNA presence in blood  of both cancer patients and healthy donors—caspase-induced DNases periodically cleave DNA within the internucleosomal linker region (the exposed DNA that is not wrapped around histone octamers [147 bp with a DNA linker of 20–90 bp, mainly 20 bp]).^75^ Despite other conflicting reports,^76^ there is now a growing body of evidence that cfDNA in cancer patients is even more fragmented compared with cfDNA from healthy donors, with a significant proportion of fragments shorter than 145 bp occurring with a 10 bp periodicity.^66,77–79^ The 10-bp periodic oscillation observed might correspond to the wrapping and protecting of the DNA from enzymatic cleavage around the nucleosome or a protein complex.^8^ Consequently, whether ctDNA is effectively shorter than nontumour cfDNA is a pivotal question. The detection of tumour-specific genetic alterations (including CNA and mutations) in human plasma and in the plasma of mice bearing human cancer xenografts revealed that mutant ctDNA is generally more fragmented than nonmutant cfDNA, with a maximum enrichment in fragments between 90 and 150 bp,^66,78^ an observation that was harnessed to enhance mutation detection using either in vitro or in silico size selection.^78^ Low-coverage WGS used to analyse the fragmentation pattern of cfDNA on a genome-wide scale showed overall that the lengths of cancer-derived cfDNA molecules were more variable than those of wild-type cfDNA, ranging from 30 bases smaller to 47 bases larger.^80^ Furthermore, the inclusion of cfDNA fragmentation in machine-learning algorithms can contribute to improving cancer detection, as the combination of cfDNA fragmentation pattern and somatic alteration analysis was shown to efficiently separate healthy subjects from cancer patients.^78,80^ In particular, this low-pass WGS approach called DELFI (DNA evaluation of fragments for early interception) is able to analyse minute amounts of cfDNA, therefore opening up new avenues for early cancer detection, especially promising because of the prevalence of clonal haematopoiesis.

Interestingly, some studies have reported the presence of large DNA fragments of several kilobases in the blood plasma of human cancer patients,^74^ but cfDNA over 350 bp was estimated to represent less than 2% of genome equivalent copy number in cancer patients.^48^ These long fragments might also indicate a necrotic, rather than apoptotic, release mechanism^66,73^ or might originate from active secretion.^81^ However, these fragments could also derive from lysed blood cells and may be a preanalytical parameter to assess as quality control of the cfDNA extract.^48^ Third-generation sequencing methods based on long reads sequencing would be helpful to investigate the biological significance of these long DNA molecules.^82^

Significantly, the fragmentation pattern of cfDNA can also be studied in other biological fluids such as urine and cerebrospinal fluid (CSF). Notably, however, a matched comparison of cfDNA in plasma, urine and CSF from glioblastoma patients revealed a different fragment distribution in CSF to that in plasma and urine, with a specific enrichment for tumour-derived cfDNA of fragments around 145 bp and a substantial proportion of fragments smaller than 145 bp.^83^ This fragmentation signature could provide an alternative way to detect the presence of ctDNA in CSF that requires no prior knowledge of point mutations or SCNAs within the tumour. The fragment distribution is also different between plasma and urine, with smaller fragments in urine centred around 82 bp.^84,85^

The ability to extract and analyse small fragments of cfDNA therefore appears to be a critically important parameter in the detection of ctDNA. Importantly, wide variability in yield and fragment size across different extraction kits has been reported, making the choice of appropriate isolation method an important analytical parameter.^86,87^ Moreover, single strand DNA template analysis revealed a higher proportion of shorter cfDNA fragments (below 80 bp) that are not readily detectable by standard double-stranded DNA library preparation protocols^48,88^ implying careful consideration when choosing the method to analyse ctDNA.

### cfDNA fragmentation in the analysis of the tissue of origin

Importantly, the results of the two large surveys of cfDNA fragmentation^78,80^ have highlighted that both the overall size distribution and the fragmentation pattern throughout the genome varies across different cancer types, suggesting the potential for cfDNA size profiling to reveal the tissue source of cfDNA.^80^ Indeed, the degree and diversity of the size fragmentation profiles reflect the different molecular structures that contain DNA (e.g. mononucleosomes, oligonucleosomes, hemi-nucleosomes, short sized transcription factors binding double strand DNA and so on) that are released from the cells and that undergo dynamic degradation in blood by endonucleases or exonucleases. Of note, the identification of the nucleases implicated in the fragmentation process in blood is still a subject of investigation.^89^ It cannot be excluded that shorter cfDNA fragments could result from the degradation in blood of longer cfDNA originating from necrosis, phagocytosis, micro-particle-containing DNA, or active release from lymphocytes.^48^ Nevertheless, nucleosome positioning, which defines DNA accessibility to nucleases, appears to play a significant role in shaping such cleavage patterns. As nucleosome positioning is an epigenetic determinant of gene expression that is cell- or tissue-specific,^90^ it has been hypothesised that the tissue-of-origin of cancer could be inferred from nucleosome positioning.

The location of nucleosomes along genomic DNA can be uncovered by cfDNA deep sequencing features such as the number and distribution of fragments or the distribution and/or orientation of their endpoints.^88,91–93^ Indeed, the number of fragments across the genome defining a depth coverage pattern reflects the nucleosome protection of DNA, and correlates with the results of nuclear chromatin micrococcal nuclease (MNase) sequencing assays in cell lines.^91,92^ In MNase assays, digestion with the endonuclease allows the periodic spacing of assembled nucleosomes to be unravelled as the enzyme preferentially cleaves the exposed internucleosomal linker region of the chromatin. Therefore, it was hypothesised that the cfDNA cleavage pattern, which retains the characteristics of chromatin structure, can be exploited to infer tissue of origin and estimate gene expression. cfDNA read depth data from the plasma of healthy donors demonstrated peak patterns that correlated closely with those found in the micronuclease map of a lymphoblastoid cell line, further confirming the consistency of nucleosome positioning between cfDNA and its cognate tissue of origin and that cfDNA shed in the bloodstream of healthy donors mainly originates from the haematopoietic system^91,92^ a finding further confirmed by a genome-wide map of nucleosome occupancy in cfDNA.^88^

Open chromatin regions are recognised as regulatory elements with well-positioned nucleosomes arrays flanking a depleted nucleosome region in the centre. This region of the chromatin is tissue specific.^94,95^ Sun et al. introduced differentially phased fragment end signals, which represent differences in the read densities of sequences corresponding to the orientation of the upstream and downstream ends of cfDNA molecules in relation to the reference genome.^93^ The quantification of differentially phased cfDNA fragment ends allowed to unravel specific fragmentation patterns within the cfDNA molecule. These cfDNA patterns were identical to nucleosomal signatures found in tissue open chromatin region. Using this analytical approach, authors could identify lymphoblastoid cells as well as the liver as important contributors to the plasma DNA pool in healthy individuals.^93^ Such a finding confirmed the hypothesis that cfDNA would only show the characteristic fragmentation patterns at open regions of chromatin where the corresponding tissues contributed DNA in the plasma. It appears that elucidating nucleosome positioning opens promising new perspectives to identify the tissue source of origin of cancer from cfDNA, with an important clinical value to classify cancers and, to a further extent, to characterise cancers of unknown origin, for example. The quantification of differentially phased cfDNA fragment ends applied to the plasma DNA from hepatocellular carcinoma and lung cancer patients correlated with the tumour DNA fraction (measured by CNA) in plasma and could identify the contribution of the corresponding tumoural tissue of origin.^93^ Using another approach, Snyder et al. showed that nucleosome spacing inferred from cfDNA could also correctly identify the contribution of tumoural lineages in cfDNA from four metastatic cancer patients who presented with a high proportion of tumour-derived cfDNA.^88^

### cfDNA fragmentation for the analysis of gene expression

It seems that cfDNA fragmentation could also reflect a general picture of gene expression. By focusing on short cfDNA fragments, Snyder et al. showed that nucleosome positioning directly harbours footprints of the in vivo occupancy of DNA-bound transcription factors.^88^ Indeed, the loss of nucleosome positioning on both sides of transcription starting sites (TSS) is necessary for proper gene expression, to create a nucleosome-depleted region over the promoter that allows transcription factors to bind. Ivanov et al. used whole-exome sequencing data to demonstrate that cfDNA coverage downstream of TSSs reflects the classic silenced and highly expressed gene patterns.^91^ The data did not cover the region upstream of TSS, including the nucleosome-depleted regions, as the capture of cfDNA during library preparation targeted only the exome and the untranslated region (UTR), enabling the prediction of expression possible for only a limited number of genes. Ulz et al., however, used whole-genome sequencing data to cover the entire promoter region in their analysis.^92^ Two different regions were identified within TSSs at which different read depth coverage patterns for expressed and silenced genes were determined by nucleosome occupancy. Accordingly, a reduction in nucleosome occupancy for expressed housekeeping genes corresponded to decreased coverage.

A key point to address would be whether cfDNA datasets from cancer patients could predict the expression of the corresponding genes in their tumours. However, this represents a challenging task due to the various proportions of DNA released from tumour and nontumour cells, and preliminary in silico simulations showed that more than 75% of cfDNA fragments for a given TSS must be released by tumour cells to be able to infer expression status. In two patients with metastatic breast cancer presenting a high proportion of ctDNA, isoforms of cancer driver genes were identified in regions with somatic CNAs from cfDNA analysis and determination of their expression was confirmed by RNA sequencing of the matching primary tumour.^92^ Fragmentation patterns from WGS data of plasma DNA have been used to infer the accessibility of transcription factor binding sites, and this approach has enabled tumour subtypes to be predicted in prostate cancer patients, as well as the detection of early stage colorectal carcinomas,^96^ emphasising the clinical potential of this minimally invasive approach. Application of this method to track and decipher tumour resistance mechanisms driven at the transcriptional level (like tumour phenotype switching upon targeted therapies or immunotherapy) would be of high interest. However, these studies traditionally require a high content of bioinformatics analysis that is not readily amenable to routine diagnosis.

## DNA methylation

Understanding how other epigenetic phenomena such as methylation patterns or histone modification can affect cfDNA fragment size could also contribute to the improved identification of cancer patients. CpG islands are regions of DNA of at least 200 bp that contain a large number of CpG dinucleotide repeats; they are usually found within the promoter region and/or within the first exon of more than 60% of human genes. Under physiological conditions, CpG islands are usually unmethylated, whereas most CpG dinucleotides outside CpG islands are methylated. During cellular transformation, however, methylation profiles are reversed, with hypomethylation of CpG dinucleotides outside CpG islands and hypermethylation of CpG islands.^97^

### Approaches to analyse methylation

In tissue, three major methods have been developed to differentiate methylated from unmethylated DNA. The most widely used technique for mapping DNA modification involves bisulphite treatment, during which unmethylated cytosine is deaminated to uracil while leaving methylated cytosine unchanged. The bisulphite-treated DNA can then be analysed by methylation-specific PCR (MSP) or sequencing, for example. Another popular method uses methylation-sensitive restriction enzymes prior to DNA amplification and detection: the methylation-sensitive enzymes digest only unmethylated CpG-containing motifs, generating digested DNA fragments that are enriched for unmethylated CpGs at their ends. Finally, affinity-enrichment-based methods have also been used in methylation status profiling. The methylated DNA immunoprecipitation (MeDIP) approach relies on anti-methylcytosine antibodies whereas a similar approach uses methyl CpG-binding-domain proteins to enrich for methylated DNA.^98,99^ All these methods can be combined with high-throughput analysis such as NGS. As such, a large number of differentially methylated genes can be identified in a single experiment.

The analysis of methylation in liquid biopsy samples from cancer patients, however, is much more challenging due to the minimal amounts of tumour-derived cfDNA in plasma.^100^ Consequently, affinity-based enrichment approaches such as MeDIP are relevant in the detection of cfDNA methylation.^101^ Despite bisulphite treatment being harmful for cfDNA, as it leads to damage and loss of the starting material, it remains the gold standard method for deciphering methylation in cfDNA. A 2019 study adapted the reduced representation of bisulphite sequencing (RRBS) method for the analysis of cfDNA methylation in liquid biopsy samples (called cf-RRBS); this approach avoids the high cost of whole-genome bisulphite sequencing (WGBS), which requires deep sequencing for a reliable cfDNA methylation analysis and is not suitable for routine use.^102^ In cf-RRBS, all ‘off-target’ cfDNA fragments not generated by the methylation-sensitive enzyme (MspI) are specifically degraded, thereby focusing the analysis on the ‘on target’ regions.

### Potential clinical application of cfDNA methylation analysis

The clinical potential of cfDNA methylation analysis in cancer has been demonstrated in numerous studies investigating mainly single gene methylation profiles in different cancer entities (reviewed extensively elsewhere in refs. ^103–105^). These studies have shown that methylated cfDNA derived from plasma or serum was associated with several clinical applications ranging from monitoring treatment and predicting response to therapy to indicating prognosis and detecting neoplastic lesions. A very recent study explored plasma methylome of metastatic castration-resistant prostate cancer patient and revealed hypomethylation of AR binding sequences associated with AR copy number gain. Patients with such methylation pattern were shown to have a more aggressive clinical course.^106^ Notably, methylation status evaluation of diverse genomic elements in cfDNA will become of high interest in the context of the emergent promising concept of epigenetic therapy combination with immune oncology drugs in the next future.^107,108^

Furthermore, other studies have generated prediction models for tumour burden based on the methylation profile of plasma cfDNA.^109^Methylation patterns are unique to each cell type and remain highly stable under physiological and pathological conditions such as cancer.^110^ As such, plasma DNA methylation analysis might have the potential to detect tissue of origin for cfDNA, thereby aiding in cancer classification and characterisation. The application is not restricted to cancer, with Poon et al.^111^ and Lun et al.^112^ reporting differential methylation in cfDNA from foetal and maternal blood during pregnancy. Similarly, Lehmann–Werman et al.^113^ used targeted sequencing of methylation-tissue-specific markers to trace back the tissue of origin of cfDNA (pancreatic β-cell DNA, oligodendrocyte DNA, neuronal/glial DNA and exocrine pancreas DNA) in plasma and thus detect cell death in specific tissues from patients with type 1 diabetes and islet-graft recipients, relapsing multiple sclerosis, traumatic brain injury or cardiac arrest, pancreatic cancer or pancreatitis, respectively. These pioneering studies opened up the field for the study of cfDNA methylation patterns for early detection of cancer. Plasma cfDNA tissue of origin mapping was also confirmed by Sun et al.^114^ while performing whole-genome-wide bisulphite sequencing (WGBS) on plasma DNA coupled with a deconvolution process to unravel the contributions of different tissue types to the plasma DNA pool, notably in the context of cancer disease.

Although promising, such studies are challenging to reproduce because of the high cost and the time-consuming nature of the genome-wide bisulphite sequencing (WGBS) technique. However, it is worth mentioning that only the relative contribution of cfDNA from different tissues is determined by methylation deconvolution based on a sequencing method and not the absolute concentration of cfDNA originating from each tissue. It would be particular interesting to ascertain the absolute concentration of cfDNA when more than one organ is suspected to release DNA, which is the case for metastatic tumours, for example. Consequently, and in order to overcome the high expenses and technical challenges that still present a hurdle in the methylation deconvolution process, digital PCR-based methods might be a solution due to their cost effectiveness and high turnaround time. Gai and co-workers developed a ddPCR assay for the detection and quantification of plasma DNA derived from the liver and the colon by targeting specific regions that are differentially methylated in the tumour-bearing tissue (liver and colon) when compared with other types of tissue.^115^ In a broader approach, Shen et al. successfully used MeDIP coupled to sophisticated bioinformatics tools to distinguish multiple types of early stage cancers with high sensitivity;^101^ this study also confirmed the consistent overlap of the epigenetic signature between the primary tumour and the plasma DNA as important prerequisite for future clinical applications of cfDNA methylation-based liquid biopsies.

## Virus-specific DNA elements

The non-human origin of viral DNA makes it a highly interesting and specific marker for monitoring virus-associated cancers using liquid biopsy samples. We now know that several different cancer types are closely linked to specific viral infections. More than 99% of cases of cervical carcinoma are attributable to human papillomavirus (HPV) infection whereas around 30% of oropharyngeal head and neck squamous cell carcinoma (HNSCC) cases are considered to be caused by persistent HPV infection. HPV comprises a large group of double-stranded DNA viruses, of which around 15 are considered high risk types, causing different squamous epithelial cancers including cervical, vaginal, vulvar, penile, anal and oropharyngeal. The double-stranded DNA virus Epstein–Barr virus (EBV) as well as persistent infections (viral and bacterial) are associated with certain cancers such as nasopharyngeal carcinoma (NPC) and gastric cancer and non-Hodgkin lymphoma in children.^116,117^

Several studies have shown that circulating viral DNA is detectable the plasma of patients with HPV-and EBV-associated cancers, with plasma HPV DNA shown to be a highly sensitive and specific biomarker, especially when detected using digital PCR-based methods.^118^ Table 2 shows studies in which the detection of circulating HPV DNA has been assessed in serum or plasma from patients with different HPV-associated cancers. Most studies on cervical cancer have involved rather small groups of patients, except for the larger 2019 study by Cheung et al., in which pretreatment blood from 138 patients with cervical cancer was analysed for the presence of HPV E7 and L1 sequences.^119^ HPV DNA was detected in 61.6% of patients, and patients with a high viral load had an increased risk of disease recurrence and death at 5 years in univariate but not multivariate analysis. Furthermore, Cocuzza et al. showed that in 34.2% of women with low grade or precancerous cervical lesions, HPV cfDNA can be detected and quantified in plasma samples, an observation that paves the way for the potential use of blood as an additional prescreening tool in parallel with cervical smears.^120^ For HNSCC, the results of larger studies have been published. In a 2018 meta-analysis of data from 600 HNSCC patients from five studies investigating circulating HPV DNA as a biomarker for disease progression, the pooled sensitivity in detecting recurrence was 54% (95% CI [confidence interval]: 32–74%) and the pooled specificity was 98% (CI: 93–99.4%), with a positive predictive value (PPV) of 93% and a negative predictive value of 94%.^121^ The data clearly indicate that circulating HPV DNA is a promising tool for surveillance in patients with HPV-associated HNSCC. Interestingly, the combined use of HPV analysis in both saliva and plasma might increase the sensitivity and specificity of the assays. Ahn et al. showed that the posttreatment HPV16 DNA status was 90.7% specific and 69.5% sensitive in predicting recurrence within 3 years in HNSCC patients when plasma and saliva results were combined.^122^ Wang et al. showed that the analysis of saliva seems to be especially sensitive in cancers of the oral cavity, whereas plasma is preferentially enriched for tumour DNA from other sites.^123^ Additional papers on saliva-based liquid biopsies have also shown promising results, especially in oropharyngeal cancer.^19,124,125^

**Table 2:** Studies measuring circulating HPV DNA in different HPV-associated cancers.

Cancer entity	Number of patients	Detection method	Detection rate	Clinical association	Reference
Anal carcinoma	57	ddPCR (HPV16)	91.1% at baseline samples, 38.9% after 5 months of chemotherapy	Residual HPV cfDNA detected at completion of chemotherapy was associated with shorter PFS and 1-year OS	^148^
Anal carcinoma	33	ddPCR (HPV16 or 18)	87.9% of stage II–III patients at baseline. After chemoradiotherapy 17%	HPV cfDNA after chemoradiotherapy was significantly associated with shorter DSF	^149^
Cervical carcinoma	138	ddPCR (E7 and L1)	61.6% at baseline	High viral load (≥20 E7 or L1 copies in 20 μL reaction volume) had increased risk of recurrence and death at 5 years	^119^
Cervical carcinoma	21	junction-specific PCR	23.9% at preoperation	HPV cfDNA significantly associated with reduced PFS	^150^
Cervical carcinoma	19	ddPCR (HPV16 and 18)	100% at baseline, 0% in healthy controls	Persistent clearance of HPV cfDNA was only observed in patients with complete response	^151^
Cervical (*n* = 47), anal (*n* = 15) oro-pharynx (*n* = 8) carcinoma.	70	ddPCR (HPV16 and 18, E7)	87% at baseline	HPV cfDNA levels in cervical cancer were related to the clinical stage and tumour size	^152^
Cervical carcinoma and dysplasia	68	PCR + RFLP	11.8%		^153^
Cervical carcinoma	16	qPCR (HPV16 and 18, E7)	81.2%	HPV cfDNA concentration in patients serum was related to tumour dynamics.	^154^
Cervical dysplasia	120	qPCR (7 HPV variants)	34.2%		^120^
HNSCC	200	TaqMan-qPCR (HPV17 and18)	14%	Baseline HPV cfDNA was associated with higher N stage and stage IV	^155^
HNSCC	47	ddPCR (HPV16 or 18)	86% at baseline	The combined saliva and plasma analysis detected in 96% HPV cfDNA	^123^
HNSCC	70	qPCR (E7)	17%		^156^
Oropharyngeal carcinoma	262	qPCR (HPV16 E6/7)	87% at baseline among HPV-pos patients, 11.5% in HPV-neg patients	Baseline HPV cfDNA was associated with higher N stage and overall disease stage.	^157^
Oropharyngeal carcinoma	93	qPCR (HPV16 E6/7)	67.3% at baseline	The combined saliva and plasma posttreatment HPV cfDNA status was 90.7% specific and 69.5% sensitive in predicting recurrence within 3 years.	^122^
Oropharyngeal carcinoma	40	qPCR (E6/7)	65% at baseline	HPV cfDNA correlated significantly with the nodal metabolic tumour volume with persistent clearance in patients with complete response	^158^

*DFS* disease-free survival, *ddPCR* droplet digital PCR, *qPCR* quantitative PCR, *HNSCC* head and neck squamous cell carcinoma, *HPV* human papilloma virus, *OS* overall survival, *PFS* progression-free survival, *RFLP* restriction fragment length polymorphism.

The role of circulating EBV in NPC has also been assessed in many studies.^126^ The presence of plasma EBV-DNA has been shown to be of clinical value in prognostication,^127,128^ monitoring of recurrence^129,130^ and even in screening for NPC.^131^ Leung et al. showed that EBV-DNA load at the midpoint of a radiotherapy course can predict outcome in NPC patients.^129^ Of the 107 patients investigated, 35 patients failed therapy; EBV-DNA was detectable in 74% of these patients. EBV detection was more predictive of outcome than was tumour stage.^129^ In another similar study of a cohort of 949 NPC patients, high EBV-DNA loads before treatment, at mid-treatment and at the end of treatment were all associated with significantly poorer overall survival, distant metastasis-free survival and progression-free survival.^132^ Recently, Lv et al. quantified cfEBV copy numbers longitudinally in 673 locally advanced nasopharyngeal carcinoma patients. The inter-patient heterogeneity in viral copy number clearance was used to define prognostic phenotypes distinguishing early, intermediate, late and no responders to chemotherapy. These data suggest that real-time monitoring of cfEBV response adds prognostic information and might have potential utility for risk-adapted treatment in NPC.^133^

A paradigm-shifting paper on the use of circulating viral DNA for NPC screening was published by Chan et al. in 2015.^131^ Of 20,000 screened asymptomatic individuals, 309 tested persistently positive for EBV, 34 of whom went on to have confirmed NPC. The sensitivity and specificity of the presence of EBV-DNA in plasma was found to be 97.1% and 98.6%, respectively. Importantly, these 34 patients were detected at earlier disease stages and thus had a better outcome than patients in historical cohorts.^131^ In order to improve the PPV for NPC screening, the same group further analysed the molecular nature of EBV-DNA in the plasma of subjects with and without NPC by target-capture sequencing and identified differences in both the abundance and size profiles of plasma EBV-DNA molecules. NPC patients had significantly more plasma EBV than disease-free patients and exhibited a reduction in the 166-bp peak (mean size of cfDNA), but showed a more pronounced peak at around 150 bp. Furthermore, compared with non-NPC subjects, NPC patients had fewer EBV-DNA molecules that were shorter than 110 bp. By combining quantitative and size-based characteristics of plasma EBV-DNA, the authors achieved a false-positive rate of 0.7% and a PPV of 19.6% using single time-point testing without the need for a follow-up blood sample.^134^ EBV infections are also associated with gastric cancer, accounting for 8–9% of all gastric cancer cases. In a 2019 large prospective study of 2760 gastric cancer patients, 52.1% (73/140) of EBV-associated gastric carcinomas had detectable EBV-DNA.^135^ Furthermore, the plasma EBV-DNA load was found to be associated with treatment response, with the load decreasing in responders but increasing with disease progression.

Taken together, the detection of viral DNA in plasma and, in certain cases, saliva in virus-associated cancer has shown a high specificity and even potential for early screening. However, many studies still lack the statistical power to detect disease recurrence, especially among cancer patients with good prognosis. Thus, large prospective studies such as those on NPC from Lo and co-workers^131^ need to be more widely performed to evaluate the clinical relevance of these liquid biomarkers in other, different tumour entities.

## Conclusions and perspectives

Increasing amounts of data have shown that it is possible to gain information beyond mutations from cfDNA obtained from the blood plasma of cancer patients, such as from the analysis of fragmentation patterns or methylation status, which are particularly informative regarding the regulation of gene expression. Human malignant tumour cells exhibit pervasive changes in DNA methylation patterns, which consequently lead to perturbations in gene expression or genomic instability. Deciphering these aberrant epigenetic modifications is of primary importance in light of the potential clinical perspectives in cancer management, ranging from early cancer detection to estimating prognosis and monitoring therapy response. Studies on cfDNA have also shown the emerging clinical potential for the early detection of virus-associated cancers, taking advantage of the lower complexity of different causative viral DNAs compared with the complex spectrum of somatic mutations in solid tumours. Nevertheless, a remaining challenge will be to distinguish transient viral infections from cancer-causing persistent infections. The detection of viral ctDNA sequences can also provide important basic information on the biology and kinetics of cfDNA in blood plasma. Serial monitoring of EBV load in plasma from NPC patients who have undergone nasopharyngectomy revealed that plasma EBV cfDNA was cleared at a rate that followed the first‐order kinetics model of decay with a median half‐life of only 139 min.^136^ The data show that the elimination of EBV-DNA is very rapid and a blood draw after surgery might be therefore an even better predictor for disease recurrence than the baseline measurement.

An important prerequisite for the introduction of the analysis of cfDNA into cancer diagnostics is the standardisation of preanalytical and analytical variables of the existing cfDNA technologies. For this purpose, international consortia including partners from academia and industry, such as CANCER-ID or the ELBS, have been established and ring experiments (same samples or methods used in parallel at several sites)—have been performed.^137^ In addition, a better understanding of the parameters that affect the release of DNA by tumour cells and host cells, as well as the effects of renal clearance, carrier proteins or extracellular vesicles in the blood plasma, thereby influencing the concentration of ctDNA and cfDNA in cancer patients,^138^ would be of great importance. Increasing data suggest that other non-blood-based liquid biopsy approaches based on e.g. saliva, CSF or urine are reliable for inclusion in future clinical trials. Finally, it should be mentioned that other liquid biopsy analytes, such as circulating tumour cells, circulating microRNAs, tumour-educated platelets or tumour-associated proteins, might provide complementary information on tumour evolution and response to therapy in cancer patients.^28,55,139^ Consequently, the development of a complex multi-analyte biomarker panel, which would require sophisticated bioinformatics tools such as machine-learning algorithms,^140^ could contribute significantly to the noninvasive management of individual patients with cancer.

To sum up, the concept of liquid biopsy introduced 10 years ago^141^ has opened new avenues in cancer diagnostics, and interventional clinical trials with established outcome measures are now needed to further demonstrate the clinical utility of ctDNA and other biomarkers.

## Data Availability

Not applicable for this review article.
